# Case–case analyses of cryptosporidiosis and giardiasis using routine national surveillance data in the United States – 2005–2015

**DOI:** 10.1017/S0950268819000645

**Published:** 2019-04-22

**Authors:** K. M. Benedict, S. A. Collier, E. P. Marder, M. C. Hlavsa, K. E. Fullerton, J. S. Yoder

**Affiliations:** 1 Waterborne Disease Prevention Branch, Division of Foodborne, Waterborne, and Environmental Diseases, National Center for Emerging and Zoonotic Infectious Diseases, Centers for Disease Control and Prevention, Atlanta, GA, USA; 2 Epidemic Intelligence Service, Division of Scientific Education and Professional Development, Center for Surveillance, Epidemiology, and Laboratory Services, Centers for Disease Control and Prevention, Atlanta, GA, USA; 3 Enteric Diseases Epidemiology Branch, Division of Foodborne, Waterborne, and Environmental Diseases, National Center for Emerging and Zoonotic Infectious Diseases, Centers for Disease Control and Prevention, Atlanta, GA, USA

**Keywords:** *Cryptosporidium*, giardiasis, surveillance

## Abstract

Understanding endemic infectious disease risk factors through traditional epidemiological tools is challenging. Population-based case–control studies are costly and time-consuming. A case–case analyses using surveillance data addresses these limitations by using resources more efficiently. We conducted a case–case analyses using routine surveillance data reported by 16 U.S. states (2005–2015), wherein reported cases of salmonellosis were used as a comparison group to identify exposure associations with reported cases of cryptosporidiosis and giardiasis. Odds ratios adjusted for age and reporting state (aOR) and 95% confidence intervals (95% CI) were calculated. A total of 10 704 cryptosporidiosis cases, 17 544 giardiasis cases, and 106 351 salmonellosis cases were included in this analyses. When compared with cases of salmonellosis, exposure to treated recreational water (aOR 4.7, 95% CI 4.3–5.0) and livestock (aOR: 3.2; 95% CI: 2.9–3.5) were significantly associated with cryptosporidiosis and exposure to untreated drinking (aOR 4.1, 95% CI 3.6–4.7) and recreational water (aOR 4.1, 95% CI 3.7–4.5) were associated with giardiasis. Our analyses shows that routine surveillance data with standardised exposure information can be used to identify associations of interest for cryptosporidiosis and giardiasis.

Cryptosporidiosis and giardiasis are nationally notifiable gastrointestinal illnesses caused by the parasites *Cryptosporidium* and *Giardia*, respectively. In the United States, an estimated 748 000 cryptosporidiosis cases and 1.2 million giardiasis cases occur annually [1]. Based on cases reported to CDC, the annual incidence rate per 100 000 population was 3.03 for cryptosporidiosis and 5.74 for giardiasis in 2015; 9735 cases of cryptosporidiosis and 14 385 cases of giardiasis were reported to CDC [2]. Our knowledge of risk factors for these diseases (https://www.cdc.gov/parasites/crypto/infection-sources.html, https://www.cdc.gov/parasites/giardia/infection-sources.html) has been informed by case–control studies of sporadic (that is, not outbreak-associated) disease [3, 4], outbreak investigations and outbreak surveillance data (https://www.cdc.gov/healthywater/surveillance/index.html). Outbreaks of cryptosporidiosis and giardiasis have been associated with an array of exposures: ingesting contaminated drinking water, exposure to treated and untreated recreational water, contaminated food and contact with ill animals (particularly pre-weaned bovine calves for cryptosporidiosis) or ill persons (particularly children).

Case–control studies are a common and preferable way to identify risk factors. However, because both cryptosporidiosis and giardiasis, such as other enteric illnesses, are under-reported to public health agencies, the source population from which the reported cases arise almost certainly differs from the characteristics of randomly-selected, illness-free general population controls which are required to produce an unbiased causal effect in case–control studies. To avoid biases introduced by these differences, researchers have suggested selecting cases of another disease from among those captured by the same surveillance system to serve as the control or comparison group to estimate differential risk [5]. Using cases of another, similar illness captured in the same surveillance system has other benefits as well, including saving time and resources needed to identify healthy controls willing to participate (e.g. through random digit dialling) and possibly reducing recall bias since those in the comparison group were also ill. Previously reported case–case analyses have used cases with different subtypes of the same illness as well as cases of other illnesses as the comparison group [6, 7]. To our knowledge, this study is the largest case–case analyses for enteric illnesses using routinely reported national surveillance data in the United States. We used the case–case analyses approach with national surveillance data to identify associations of interest for sporadic cryptosporidiosis and giardiasis in the United States. We used reported cases of salmonellosis as the comparison group for reported cases of cryptosporidiosis and giardiasis.

Cases of cryptosporidiosis, giardiasis and salmonellosis are reported to the National Notifiable Diseases Surveillance System (NNDSS) (https://wwwn.cdc.gov/nndss/document/NNDSS-Fact-Sheet-508.pdf) at CDC using national case definitions developed by the Council of State and Territorial Epidemiologists (CSTE) (https://wwwn.cdc.gov/nndss/case-definitions.html). Cases that met the case definition at the time of transmission were included in this analyses. State health departments collect exposure information based on standardised questionnaires in each state and report it to CDC. Health providers, hospitals or laboratories are mandated to report certain diseases to the states, then state health departments notify CDC on a voluntary basis. States routinely transmit reports of cases and related epidemiological, laboratory and limited exposure data to NNDSS using integrated surveillance information systems in public health departments. These systems are based on the National Electronic Disease Surveillance System (NEDSS) standards; states either use a NEDSS Base System (NBS), developed by CDC or a NEDSS compliant system (https://wwwn.cdc.gov/nndss/nedss.html) [8]. Only states using NBS systems routinely transmit exposure data to CDC; data from these states were used in this analyses: Alabama, Arkansas, Idaho, Maine, Maryland, Montana, Nebraska, Nevada, New Mexico, Rhode Island, South Carolina, Tennessee, Texas, Vermont, Virginia and Wyoming. The remainder of states were not using NBS at the time of this analyses and therefore were not routinely transmitting exposure data to NNDSS.

While cryptosporidiosis, giardiasis and salmonellosis were nationally-notifiable for the entire 11-year timeframe of these analyses, each state has its own laws and regulations defining what diseases are reportable (https://wwwn.cdc.gov/nndss/data-collection.html); giardiasis was not reportable in Texas during 2008–2015, Vermont during 2015 and Tennessee during 2010–2015 [2]. Case-patients that reported recent international travel or were specifically associated with an outbreak were excluded from this analyses, but those missing information on international travel or outbreak status were included. For these analyses, it was assumed that cryptosporidiosis, giardiasis and salmonellosis case-patients were asked about exposures within the same timeframe.

These analyses included 19 exposure variables which were collected and labelled in common across reported cryptosporidiosis, giardiasis and salmonellosis cases, and which were transmitted to and available for analyses at CDC (data accessed in December 2016). Four variables with ⩾70% missing data for any single pathogen were excluded; i.e. new pet, school/work water source, home well treatment and school/work well treatment. Cases with a reported exposure were coded as exposed and all other cases, including cases with missing information for that exposure, were coded as not exposed to include all cases in model execution (except for those with missing state or age information). High-level categories of exposures for analyses were created from the reported exposure variables. Animal contact exposures were categorised as follows: livestock, reptile/amphibian, poultry and dogs. Recreational water exposures were categorised as treated or untreated recreational water. Referent groups were selected as ‘no exposure’ group whenever possible; the only exception was for the home water source exposures, where municipal water as a source serves as the referent group. Percentages were calculated among all cases; therefore, cases with missing exposure, age or reporting state information were included in the denominator. For the case–case analyses, reported cryptosporidiosis and giardiasis cases were compared with reported salmonellosis cases. Odds ratios adjusted for age and reporting state (aOR) and 95% confidence intervals (95% CI) for each exposure were calculated with binomial logistic regression using SAS 9.4. We chose not to attempt adjusting for additional confounders (e.g. sex), building multivariable models of exposures, or calculating population attributable fractions due to the large proportion of missing data in these routine surveillance data.

A total of 10 704 cryptosporidiosis, 17 544 giardiasis and 106 351 salmonellosis domestically-acquired sporadic cases were reported to CDC from NBS-states during the study period. The count of reported cases varied across the states for cryptosporidiosis cases (73 in Nevada to 3522 in Texas) and giardiasis cases (209 in Wyoming to 3096 in Virginia). Both cryptosporidiosis and giardiasis displayed bimodal age distributions, with the highest proportions of reported cases in those aged 0–9 years (cryptosporidiosis: 27.6%, giardiasis: 23.0%) and adults aged 30–44 years (cryptosporidiosis: 18.2%, giardiasis: 21.4%).

When adjusting for reporting state and age and in comparison with reported salmonellosis, reported cryptosporidiosis was associated with livestock, multiple person-to-person transmission exposures and multiple water exposures (Table 1). The highest odds ratios for reported exposures associated with cryptosporidiosis compared with salmonellosis included livestock contact (aOR 3.18, 95% CI 2.87–3.52) and overall recreational water exposure (aOR 4.59, 95% CI 4.30–4.91). Reported exposure to treated recreational (aOR 4.66, 95% CI 4.30–5.04) and untreated (aOR 3.85, 95% CI 3.48–4.26) recreational water was significantly associated with reported cases of cryptosporidiosis when compared with salmonellosis.

**Table 1.** Exposure variables investigated for potential associations of interest for cryptosporidiosis (*n* = 10 704) and giardiasis (*n* = 17 544) compared separately with salmonellosis (*n* = 106 351) using routine surveillance data from 16 states, 2005–2015

	Cryptosporidiosis				Giardiasis				Salmonellosis	
	No.	%^a^	aOR^b^	(95% CI)	No.	%^a^	aOR^b^	(95% CI)	No.	%^a^
Animal contact^c^	3766	35.2	1.11	(1.05, 1.17)	3960	22.6	0.83	(0.79, 0.87)	29 341	27.6
Livestock	732	6.8	3.18	(2.87, 3.52)	255	1.5	0.97	(0.83, 1.13)	1482	1.4
Reptile/amphibian	100	0.9	0.27	(0.22, 0.33)	90	0.5	0.18	(0.14, 0.23)	2773	2.6
Poultry	256	2.4	0.92	(0.80, 1.07)	167	1.0	0.50	(0.42, 0.60)	1799	1.7
Dog	2695	25.2	1.10	(1.04, 1.16)	3017	17.2	0.95	(0.90, 1.00)	21 659	20.4
*Missing/not specified*	4200	39.2			10 200	58.1			52 571	49.4
Associated with childcare^d^	892	8.3	1.58	(1.46, 1.72)	938	5.4	1.31	(1.20, 1.44)	5652	5.3
*Missing/not specified*	4255	39.8			9917	56.5			53 281	50.1
Attends childcare^d^	586	5.5	1.22	(1.11, 1.35)	584	3.3	1.00	(0.90, 1.11)	4766	4.5
*Missing/not specified*	3734	34.9			9185	52.4			47 754	44.9
Lives with childcare attendee^d^	358	3.3	2.35	(2.06, 2.68)	404	2.3	1.82	(1.59, 2.08)	1349	1.3
*Missing/not specified*	4286	40.0			9787	55.8			53 185	50.0
Works at childcare^d^	89	0.8	3.11	(2.35, 4.11)	98	0.6	2.43	(1.84, 3.22)	214	0.2
*Missing/not specified*	3768	35.2			9249	52.7			47 493	44.7
Know other ill persons	1635	15.3	2.32	(2.18, 2.48)	1482	8.5	1.13	(1.05, 1.22)	7164	6.7
*Missing/not specified*	3956	37.0			9436	53.8			50 197	47.2
Drink untreated water	353	3.3	2.93	(2.54, 3.37)	568	3.2	4.07	(3.56, 4.65)	851	0.8
*Missing/not specified*	4970	46.4			11 571	66.0			60 921	57.3
Home water source										
Municipal	5210	48.7	1		6258	35.7	1		43 594	41.0
Private well	1184	11.1	1.38	(1.27, 1.49)	1202	6.9	0.97	(0.90, 1.05)	6165	5.8
*Missing/not specified*	4310	40.3			10 084	57.5			56 592	53.2
Recreational water exposure	1944	18.2	4.59	(4.30, 4.91)	1588	9.1	2.73	(2.53, 2.93)	4844	4.6
Treated	1214	11.3	4.66	(4.30, 5.04)	605	3.5	1.74	(1.56, 1.93)	2973	2.8
Untreated	749	7.0	3.85	(3.48, 4.26)	970	5.5	4.08	(3.69, 4.51)	1616	1.5
*Missing/not specified*	2831	26.5			7086	40.4			39 672	37.3

a Percentages were calculated among all cases. Therefore, cases with missing exposure information, age and reporting state were included in the denominator.

b aOR: odds ratio adjusted for age and reporting state (incidence rates by state for cryptosporidiosis [17], giardiasis [18] and salmonellosis [19] are reported elsewhere; number of cases reported 2005–2015 with Cryptosporidiosis: Alabama 583, Arkansas 345, Idaho 686, Maine 220, Maryland 361, Montana 468, Nebraska 1059, Nevada 73, New Mexico 729, Rhode Island 128, South Carolina 572, Tennessee 745, Texas 3522, Vermont 428, Virginia 630, Wyoming 155; Giardiasis: Alabama 1463, Arkansas 882, Idaho 1326, Maine 1252, Maryland 1881, Montana 695, Nebraska 1554, Nevada 211, New Mexico 649, Rhode Island 595, South Carolina 1184, Tennessee 997 (giardiasis not reportable 2011–2015), Texas n/a (giardiasis not reportable), Vermont 1550 (giardiasis not reportable in 2015), Virginia 3096, Wyoming 209; Salmonellosis: Alabama 7938, Arkansas 5199, Idaho 1349, Maine 640, Maryland 6752, Montana 873, Nebraska 2276, Nevada 472, New Mexico 2537, Rhode Island 905, South Carolina 13 710, Tennessee 8600, Texas 43 863, Vermont 661, Virginia 10 157, Wyoming 424). For modelling, variables were recoded. Cases with a reported exposure were coded as exposed and all other cases, including cases with missing information for that exposure, were coded as not exposed to include all cases in model execution.

c Exposure categories are not mutually exclusive; sum of percentages might not equal 100%.

d Reported as discrete variables. State health department may variably populate the information that informs these variables; e.g. they may ask the questions differently. We have interpreted ‘associated with childcare’ to represent cases reporting any association with childcare which could include situations included in the other categories or additional relationships with childcare not captured by the other three variables.

Similar to cryptosporidiosis, giardiasis was associated with multiple person-to-person transmission exposures and multiple water exposures, when compared with salmonellosis and adjusting for age and reporting state (Table 1). The highest odds ratios in this analyses for reported exposures associated with giardiasis were for untreated water, both drinking (aOR 4.07, 95% CI 3.56–4.65) and recreational (aOR 4.08, 95% CI 3.69–4.51) exposures. Because all the associations discussed above are known to be non-protective for salmonellosis based on case–control data [9], we can conclude from this case–case study alone that these likely represent risk factors for either cryptosporidiosis or giardiasis in the general population.

These analyses are the first, to our knowledge, to use routinely collected national enteric disease surveillance data in the United States in a case–case analyses approach to identify associations of interest. While this analyses did not identify novel exposures for either cryptosporidiosis or giardiasis, it is comforting that our findings were consistent with previously identified exposures for these illnesses using national surveillance data (https://www.cdc.gov/parasites/crypto/infection-sources.html and https://www.cdc.gov/parasites/giardia/infection-sources.html) [2, 3]. Cryptosporidiosis and giardiasis are well-documented waterborne diseases, so it follows that the exposures identified as associated with illness in this study with the greatest odds ratios were water related. This method provides benefits for the ongoing monitoring of associations of interest for cryptosporidiosis and giardiasis and health trends in the United States. By using NNDSS data, this method uses an existing public health surveillance system by which data are already transmitted from states to CDC. This allowed us to analyse a large set of data across multiple states to identify associations of interest for these nationally notifiable diseases for which it is not feasible to conduct a national-level study using the more preferable epidemiologic designs (i.e. case–control or cohort study). Although case–control studies are generally recognised as less resource intensive than other epidemiological study designs such as cohort studies, national-scale case–control studies are resource intensive and identifying enough cases and controls across the United States to detect statistically significant associations is difficult when national-level resources dedicated to cryptosporidiosis and giardiasis are limited.

The higher odds ratio for exposure to treated recreational water than untreated recreational water among cryptosporidiosis cases compared with salmonellosis is expected given the extreme chlorine tolerance of *Cryptosporidium*. Person-to-person transmission-related associations, as highlighted by attendance at childcare and contact with other similarly ill persons, were significant for both cryptosporidiosis and giardiasis, a finding observed in previous studies [3, 4]. Contact with different animals was found to be associated with each pathogen (i.e. exposure to livestock with cryptosporidiosis and exposure to dogs with giardiasis). Although livestock contact is well documented for cryptosporidiosis, the documented evidence is less clear regarding animal associations with giardiasis [10]. Further, since these comparisons were with salmonellosis, it is important to interpret these animal exposures as more associated with each pathogen than with salmonellosis.

Surveillance data, as the central nervous system of public health, are a readily available and routinely collected source of data that can be used to investigate some of the same questions as case–control or cohort studies, but in a less-resource intensive way that leverages data already being collected by states. Identification of exposures that are significantly associated with illness through case–case analyses using surveillance data is a promising use of existing data on these nationally notifiable diseases. However, our analyses were limited to a subset of states that routinely transmitted exposure data to CDC, and then limited further to only that exposure information that was collected in common across diseases. Standardised exposure information (i.e. content and common exposure windows) collected across pathogens would increase the utility of NNDSS data to identify exposures associated with illness and formulate targeted prevention measures. Continued investment is needed to modernise our national surveillance system to support efficient collection and transmission of epidemiological information [11]. More importantly, state and local health departments also require dedicated resources for investigation and reporting of nationally notifiable diseases, including collection of exposure information. Although known risk factors for cryptosporidiosis and giardiasis differ from known risk factors for salmonellosis, the collection of the same exposure information across pathogens with routine surveillance data will facilitate case–case analyses to identify pathogen-specific associations among reported cases as has been done with other pathogens [5, 12–15].

These analyses are subject to several limitations. As noted above, these data are not nationally representative, given the requirement of this analyses approach for exposure information collected in common across the pathogens that was also transmitted to NNDSS. However, the states included (those using NBS systems at the time of the analyses) were not just in one geographic region of the country so a variety of regions were included in the catchment area of these data. Second, case definitions for these diseases changed over the study period and no attempt was made in this analyses to standardise the case definitions. Third, the exposure data transmitted within these routine surveillance data is collected by jurisdictions in varying ways (i.e. questions and procedures for interviewing cases might differ from state-to-state or within state). For example, cryptosporidiosis and giardiasis have a longer incubation period than salmonellosis, and exposure periods assessed for our parasites were likely equal to or longer than that for salmonellosis. This can be a substantial source of bias including a reversal of the estimated association in some cases [16]. Fourth, a high degree of missing or not specified information was generally lowest for cryptosporidiosis and highest for giardiasis which may reflect differences with how giardiasis cases, and related exposure information, are reported in the United States. Additionally, health care providers or laboratories who report cases to the state may not collect information in a systematic way. Specifically, we believe that there is generally less follow-up on giardiasis cases than cryptosporidiosis cases at the state and local level given competing public health concerns and limited capacity. Fifth, exposures associated with the illness (e.g. cryptosporidiosis or giardiasis) in these case–case analyses should be interpreted as differential risk [5, 12–15].

When national surveillance data for nationally notifiable diseases includes standardised exposure information across states it can be used to identify associations of interest for illness by using the case–case analyses approach. In order to be a feasible approach for ongoing identification and monitoring of changes in the epidemiology of these diseases, national surveillance requires a multi-jurisdictional approach to defining, collecting and reporting common exposures for sporadic disease. Routine use of this approach could allow for efficient hypothesis generation about the causes of illness, and in some limited cases the identification of risk factors for disease, leading to improved understanding of disease epidemiology and guidance for development of prevention and control strategies.
